# Emerging role of nucleic acid aptamers in sepsis-induced coagulopathy: Future perspectives in diagnostics and therapeutics

**DOI:** 10.1016/j.aicoj.2025.100014

**Published:** 2026-01-19

**Authors:** Maeva Martin, Marine Tschirhart, Cyril Auger, Florence Toti, Julie Helms, Laurence Choulier

**Affiliations:** a University of Strasbourg, INSERM (French National Institute of Health and Medical Research), Regenerative Nanomedicine (RNM) UMR 1260, FMTS, CRBS, 1 rue Eugène Boeckel, F-67000 Strasbourg, France; b University of Strasbourg, CNRS, Laboratory of Bioimaging and Pathologies UMR 7021, Faculty of Pharmacy, 74 route du Rhin, F-67400 Illkirch, France; c Université de Strasbourg, Faculté de Médecine. Fédération Hospitalo-Universitaire (FHU) TARGET. Hôpitaux Universitaires de Strasbourg, Service de Médecine Intensive-Réanimation, Nouvel Hôpital Civil, Strasbourg, France

**Keywords:** Disseminated intravascular coagulation, Coagulopathy, Sepsis, Aptamers, Fibrinolysis

## Abstract

Sepsis-induced coagulopathy is a severe complication of sepsis and septic shock, contributing significantly to organ dysfunction and increased mortality. Currently, there is no effective treatment for coagulopathy, highlighting a critical need for new therapeutic options to improve patient outcomes. This review explores the potential use of nucleic acid aptamers for septic-induced coagulopathy.

Aptamers are short single-stranded oligonucleotides, known for their high affinity and specificity towards targets. They offer several advantages over antibodies, including smaller size, synthetic production, lower immunogenicity, and greater flexibility in targeting a wide range of molecules and cells. Due to these properties, aptamers have emerged as promising tools for the diagnosis and treatment of various diseases, with several currently ongoing clinical trials and others already available on the pharmaceutical market.

This illustrated and thoroughly documented review provides a comprehensive overview of the key aspects of sepsis pathophysiology, emphasizing the significant roles aptamers can play, including: the targeting of endothelial dysfunction where aptamers could protect vascular integrity and inhibit leukocyte adhesion to the endothelium thereby limiting local inflammation; the targeting of dysregulated coagulation pathways by preventing platelet adhesion and aggregation, as well as blocking coagulation factors, helping to limit thrombus formation; the restoration of fibrinolytic function by targeting direct inhibitors of plasmin generation; the targeting of neutrophil extracellular traps to decrease excessive coagulation activation and restore fibrinolysis; the inhibition of complement system in sepsis-induced disseminated intravascular coagulation. This review also includes a chapter on the use of aptamers as diagnostic and prognostic tools for sepsis-induced coagulopathy.

## Introduction

Sepsis is defined as life-threatening organ dysfunction caused by a dysregulated host response to infection, and may progress to septic shock, a severe form characterized by circulatory and metabolic abnormalities [1]. In 2020, the WHO identified sepsis as a global health priority, with 49 million cases and 11 million deaths reported in 2017 [2].

Sepsis can be triggered by various pathogens (bacteria, viruses, fungi, parasites), which activate local pro-inflammatory and procoagulant responses. The interaction between immunity and coagulation, termed immunothrombosis, helps contain infection through innate immune cell recruitment [3,4]. However, in sepsis, this process becomes dysregulated, promoting endothelial damage and excessive coagulation. Hyperinflammation is further amplified by complement activation and coagulation imbalance [5]. This may lead to coagulopathy and disseminated intravascular coagulation (DIC), marked by widespread microthrombosis and organ dysfunction, with mortality rates up to 65% in septic DIC [6]. Sepsis-induced DIC is also characterized by impaired fibrinolysis, partly due to neutrophil extracellular traps (NETs)-induced plasminogen degradation and endothelial release of plasmin inhibitors.

There is currently no specific treatment for sepsis-induced DIC. Management is limited to infection control and organ support, and no validated markers exist for risk stratification. In sepsis-induced DIC, nucleic-acid aptamers represent promising tools for both improving early diagnosis and enabling targeted therapy, which could potentially minimize off-targets and adverse effects of potential treatments.

This review highlights potential aptamer-based strategies for sepsis-induced coagulopathy, with focus on endothelial dysfunction, coagulation imbalance, hypofibrinolysis, and diagnosis.

## Pathophysiology of septic DIC

Since sepsis results from a dysregulated host response to infection, the first step involves immune system activation via interactions between pathogen-associated molecular patterns (PAMPs) and pattern recognition receptors (PRRs). Monocytes and neutrophils then release pro-inflammatory cytokines and procoagulant microvesicles. This hyperinflammatory state contributes to endothelial glycocalyx degradation, increasing permeability and exposing the endothelial surface to procoagulant stimuli. Damaged endothelial cells release damage-associated molecular patterns (DAMPs), amplifying systemic inflammation.

Inflammatory cytokines also upregulate tissue factor (TF) on immune and endothelial cells, initiating the extrinsic coagulation pathway. Endothelial activation further promotes immune cell adhesion and secretion of von Willebrand factor (vWF), facilitating platelet aggregation and thrombus formation. This process, known as immunothrombosis, helps trap and neutralize pathogens within the thrombus [7].

**Fig. 1:**
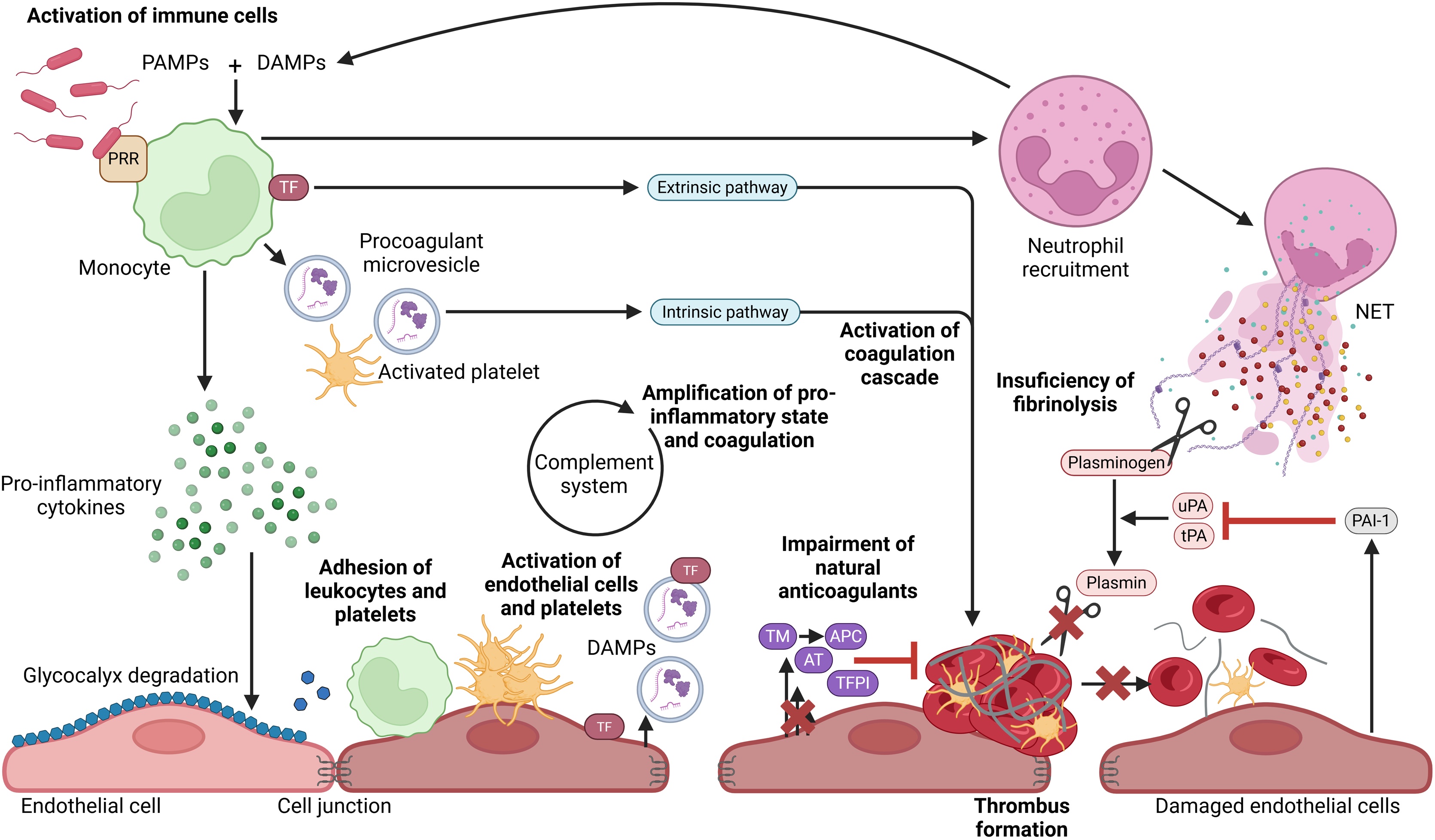
**Physiopathology of septic DIC.** Activated immune cells release cytokines which leads to the degradation of the endothelial glycocalyx, contributing to endothelial cell activation and leukocytes and platelet adhesion on endothelium. Coagulation cascade, which is activated by phosphatidylserine exposed on microvesicles, activated platelets and TF expression on immune and endothelial cells, results in microthrombus formation. Furthermore, pro-inflammatory state and damaged endothelial cells are associated to downregulation of endogenous anticoagulants and to decrease of fibrinolysis. Additionally, complement system interacts with coagulation cascade to activate each other and amplify pro-inflammatory state by its anaphylatoxin release. PAMP: pathogen associated molecular pattern, DAMP: damage associated molecular pattern, PRR: pattern recognition receptor, TF: tissue factor, NET: neutrophil extracellular trap, AT: antithrombin, TM: thrombomodulin, APC: activated protein C, TFPI: tissue factor pathway inhibitor, uPA: urokinase plasminogen activator, tPA: tissue-type plasminogen activator, PAI-1: plasminogen activator inhibitor type-1.

In septic DIC, immunothrombosis becomes dysregulated, with excessive coagulation, reduced anticoagulants (antithrombin, thrombomodulin), insufficient TF pathway inhibition, and defective fibrinolysis [[8], [138]]. Procoagulant microvesicles and increased TF expression drive clot formation, while fibrinolysis is impaired by reduced plasmin generation—due to endothelial-derived plasminogen activator inhibitors and NET-mediated plasminogen degradation [9]. NETs are DNA-based structures released during NETosis, a specific form of neutrophil cell death. Complement activation further amplifies inflammation and coagulation by releasing anaphylatoxins and enhancing TF expression, in part via a self-amplification of the complement system [8]. These mechanisms are summarized in Fig. 1.

Considering this complexity, innovative tools are needed to both better characterize and modulate these pathways. Among them, nucleic-acid aptamers have emerged as promising candidates due to their ability to specifically bind and modulate diverse molecular targets implicated in sepsis-induced coagulopathy.

## Nucleic acid aptamers

Naturally occurring nucleic acid aptamers, known as riboswitches, are RNA elements that regulate gene expression by binding specific metabolites or ions within cells. They illustrate the intrinsic ability of nucleic acids to form complex three-dimensional structures capable of high-affinity molecular recognition. However, in the context of this review, the focus will be on synthetic aptamers. They are short single-stranded DNA or RNA sequences that act as high-affinity and high-specificity ligands for a wide variety of targets. These targets range in nature and size—from small molecules (metabolites, toxins, drugs) to macromolecules (proteins, lipids, nucleic acids), pathogens (viruses, bacteria), organelles, and even whole cells [10]. This versatility distinguishes aptamers from antibodies, especially since they can also target non-immunogenic or toxic molecules [11].

**Fig. 2:**
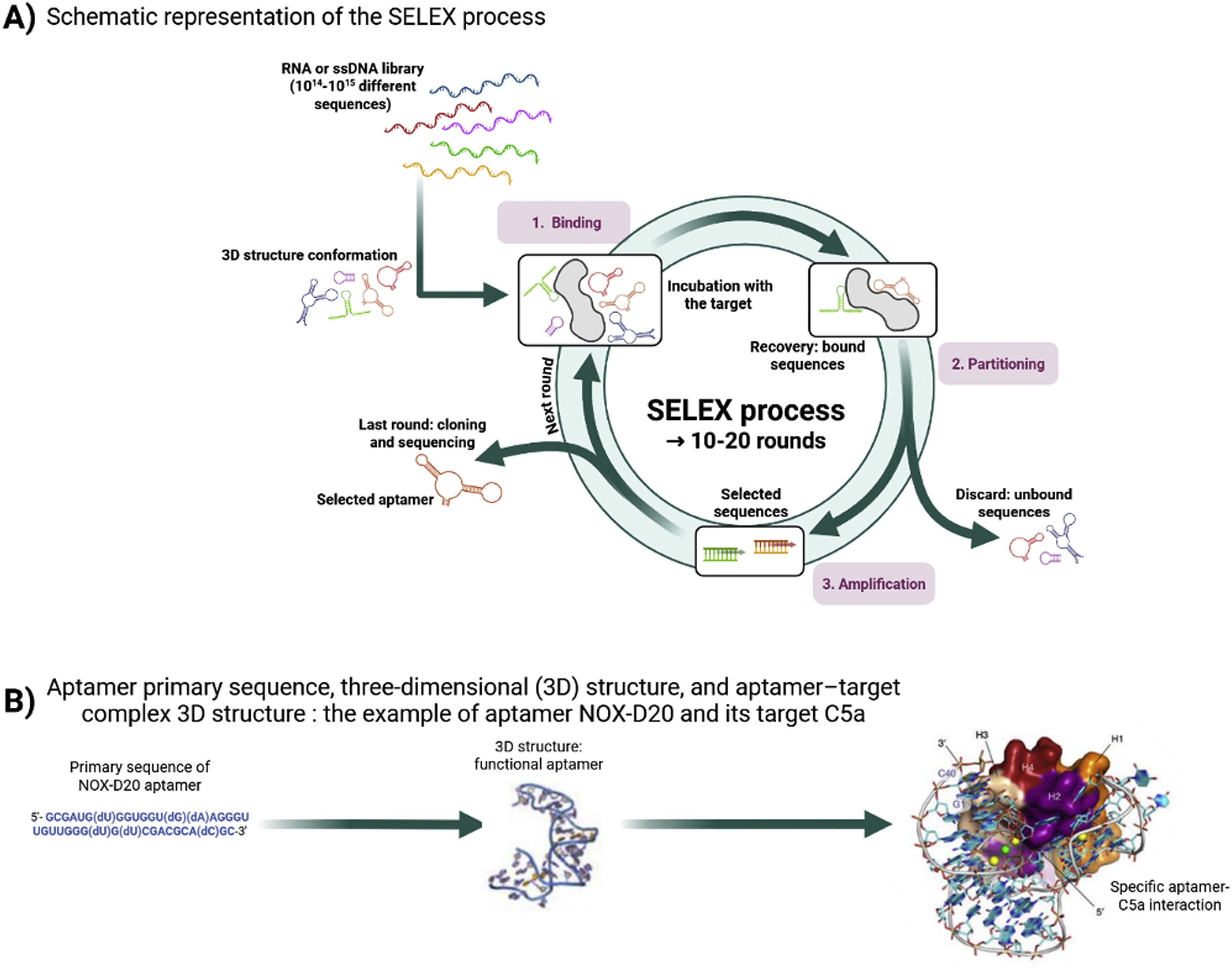
**From SELEX to aptamer–target recognition. (A)** Schematic representation of the SELEX (Systematic Evolution of Ligands by Exponential Enrichment) process leading to the selection of high-affinity nucleic acid ligands. Briefly, the nucleic acid library is incubated with the target of interest. During the partitioning step, bound and unbound fractions are separated. The bound fraction is amplified to obtain an enriched pool for next round of selection. This round is repeated between 10 and 20 times to enrich the sequences that specifically bind the target. At the last round, nucleic acid sequences, named aptamers, are cloned and sequenced. **(B)** The example of NOX-D20 aptamer and its target C5a: aptamer primary sequence, three-dimensional (3D) structure, and aptamer-target complex 3D structure [137].

Aptamers are identified through an *in vitro* selection method called SELEX (Systematic Evolution of Ligands by EXponential enrichment) [12,13]. Starting from a vast random library (∼10^15^ oligonucleotide sequences), SELEX combines repeated cycles of target binding, separation of bound from unbound sequences, and amplification to enrich for high-affinity ligands (Fig. 2). Several SELEX variants have been developed to enhance aptamer performance under physiological or complex conditions, thereby improving affinity and specificity.

Compared to antibodies, aptamers offer major advantages in terms of chemical synthesis, thermal stability, and flexibility. They have distinctive pharmacokinetic and structural properties that confer them both advantages and limitations, which can be modulated through diverse chemical and structural modifications to enhance their stability and bioavailability for therapeutic use (Table 1).

**Table 1:** Characteristics of aptamers versus antibodies. Improvement strategies are shown as footnotes.

Property	Aptamer	Antibody
Chemical property	Nucleotides	Amino acids
Specificity and affinity	High	High
Time needed for selection	Several weeks	Several months
Cost of selection	≈$ 4,000 for individual sequences	≈$ 8,000 for mouse monoclonal antibody ≈$ 20,000 for rabbit monoclonal antibody
Synthesis and manufacture	Chemical synthesis High scale production Chemical modifications	Produced in animal or by recombinant methods
Modification	Easy and controllable	Limited and uncontrollable
Storage and stability	Stable under different conditions Can recover their native structure after denaturation	Requires special conditions for storage and handling
Enzymatic degradation	Nuclease degradation (mainly for RNA)^1^	Little degradation
Batch-to-batch variations	Little or no	Difficult to avoid
Size	5−30 kDa allows rapid diffusion and increased tissue penetration^2^	Whole size of antibody is 150 kDa
Target range	Large: small molecule, protein, cell…	Limited to antigenic targets
*In vivo* complications	No evidence of immunogenicity or toxicity Immune response to (modified) aptamers is theoretically possible, but has not been described to date.	May lead immune response
Clinical application	Still immature	Mature

Improvement strategies: ^1^Chemical modifications: Sugar modifications (e.g., 2’-fluoro, amino, or methyl substitutions), Backbone modifications, such as replacing phosphodiester linkages with triazole groups (via click chemistry) or using phosphonothioate bonds, Base modifications (e.g., C5 of pyrimidines or N7 of purines), Circularization of aptamers, Spiegelmers: L-enantiomeric versions of natural D-aptamers. ^2^.To limit rapid renal clearance: Terminal modifications at the 3’ or 5’ ends with carriers (e.g., with biotin, cholesterol, or polyethylene glycol [PEG], poly(lactic-co-glycolic acid) [PLGA], or proteins), Multimerization.

Another distinctive feature of aptamers is the possibility to reverse their effect using specific antidotes—complementary oligonucleotide strands that competitively bind to the aptamer and block its interaction with the target [14]. This controllability is particularly attractive for therapeutic use. Aptamers also show promise in pathogen detection, with high sensitivity and specificity [[15], [16], [17]], and in anti-infective strategies, where they could help overcome antimicrobial resistance [18].

In the following sections, we will explore how aptamers may be harnessed to target the key pathological features of sepsis-induced coagulopathy (Table 2) — namely endothelial dysfunction, coagulation imbalance and hypofibrinolysis, as well as used as diagnostic and prognostic tools (Table 3).

**Table 2:** Summary table of aptamers with potential therapeutic effects in sepsis-induced coagulopathy.

Name	Type	Sequence 5’ → 3’	Target	Level of study	Possible effect on septic DIC	Reference
1.5M Short	DNA	ACTTTTGAATGTGGCAACAAATTGACAGG	heparanase	*In vitro*	Preserve endothelial glycocalyx	(20)
s5	DNA	ACCGGGCGTACACCGTCGCGGCACATGTCTGAATGCGTTTAGTCTCTGTG	CD44	*In vitro*	Maintain low vascular permeability	(21)
D-10-3	DNA	AGAGACCCTGACTGCGAACTCCCACTTTACCTCTTCCCTCAACTTCCCCTTCCTTTCTGACCCTGGACACGGTGGCTTCTT	ICAM-1	*In vitro*	Limit leukocyte adhesion and transmigration	(26)
BD-10-1	DNA	AGAGACCCTGACTGCGAACATATACAGACGGTAGCGACTGGAAGAGAGCCTTAACCACACATCTGGACACGGTGGCTTCTT				
B-10-4	DNA	AGAGACCCTGACTGCGAACACACATGGAACGCAACACCCTGAAACTATGGACTGACGAGACCATGGACACGGTGGCTTCTT				
B-10-6	DNA	AGAGACCCTGACTGCGAACCAACCACAGCTTAATATCCTCATATCCGCGACCGCTCGCGAGCATGGACACGGTGGCTTCTT				
VCAM-1 aptamer	RNA	AGGGAAUCUUGCCUAGGGAGGGAGUAGCGAAAGGGCUCA	VCAM-1	*In vivo*	Limit leukocyte adhesion and transmigration	(27)
D20	RNA	UGCGCGUCCCAUGGGGUAUAGAGGGGUCGAAGUGGACGCG	LFA-1	*In vitro*	Reduce cell adhesion to ICAM-1	(28)
D28	RNA	UCCUCGCUCAUACAACGAGGGGUCGUGUAGGGAUGUAUGGG				
D31	RNA	GGGCGCUAAGUCCUCGCUCACAAGGUGCAAUGCAAUAUGUGAGUGCGCCGCCCUUUCUCUCGCGCGACUCGGACCUAC				
ARC5690	RNA	40 kDa PEG – CUCGCAGACAACCGGAUGAAAUCCGACCGGAG - idT	P-selectin	*In vivo*	Limit leukocyte adhesion and rolling	(29)
SDA	DNA	GCCTGTTGTGAGCCTCCTAACGATTTGGATTTGGGGTGGAGGGTATGGTTTGTGCTGGCGTTCTCATTTCCCATGCTTATTCTTGTCTCCC	E- and P-selectin	*In vitro*	Limit leukocyte adhesion and rolling	(30)
Aptamer 1	DNA	CATGCGCCTTCCCCCTGTGGTTGGTGTCAGTCGGCCTGTGTGATTAGTGGAATGTGCGATGGGTCTCCCAGCGGGATTGG	Fibronectin	*In vitro*	Limit adhesion of platelets to the sub-endothelium	(32)
ARC1779	DNA/RNA	20 kDa PEG – GCGUGCAGUGCCUUCGGCCGTGCGGTGCCUCCGUCACGC – idT 2-O-Methyl nucleotide	vWF	Phase II	Reduce platelet aggregation	NCT00726544 NCT00632242 NCT00694785 NCT00742612 NCT00507338 NCT00432770
BT200	RNA	40 kDa – GCCAGGGACCUAAGACACAUGUCCCUGGC – idT 2-O-Methyl nucleotides		Phase II		NCT04677803 NCT04103034
ARC15105	RNA	40 kDa PEG – NH -GGGACCUAAGACACAUGUCCC-idT 2-O-Methyl nucleotides		*In vivo*		(41)
TAGX-0004	DNA	CGCGGCCGADsGAGCACCGAAGGTCTCAACGDsTGGAGGCCGCGCGCGT’AGCG		*In vitro*		(42)
R9.3	DNA	ATCGCGCTCTCCTGCTTAAGCAGCTATCAAATAGCCCACT		*in vitro*		(43)
R9.14	DNA	TGGACGAACTGCCCTCAGCTACTTTCATGTTGCTGACGCA				
DTRI-031	RNA	ACGAACUGCCCUCGAUCGACGCACAGACGUUUUUU 2-O-Methyl nucleotides *2’-Fluoro nucleotides*		*In vivo*		(44)
RB571	RNA	*29-nucleotide modified RNA however no more information is given*	GPVI	*In vitro*	Inhibits collagen-related peptide mediated platelet aggregation	(45)
R4cXII-1	RNA	GGGAGGACGAUGCGGCCAAAUCUCGGCUGCCAGCAGGUCACGAGUCGCAAGAUAACAGACGACUCGCUGAGGAUCCGAGA *2’-Fluoro nucleotides*	FXII	*In vitro*	Prolong blood clot formation	(48)
FELIAP	DNA	AACCTATCGGACTATTGTTAGTGATTTTTATAGTGT	FXIa	*In vitro*	Extend clotting times and inhibit thrombin generation	(49)
11.16	RNA	CCAGUCCGCAUCCAUCAUCCCCCUCCCCC		*In vitro*		(50)
12.7	RNA	UAACGCCACGCUCGACAACGCGUCGAGUGUCCUCCGCCCC				
REG1: RB006 (drug) / RB007 (antidote)	RNA	RB006: 40 kDa PEG – NH – GUGGACU AUACCGCGUAAUGCUGCCUCCAC – idT RB007: CGCGGUAUAGUCCCCAU 2-O-Methyl nucleotides *2’-Fluoro nucleotides*	FIXa	Phase III	Reduce thrombin generation	NCT01848106 NCT00715455 NCT00932100 NCT00113997 NCT01872572
DTRI-178 (RB006)	RNA	40 kDa PEG – NH – GUGGACUAUA CCGCGUAAUGCUGCCUCCAC – idT 2-O-Methyl nucleotides *2’-Fluoro nucleotides*		*In vivo*		(54)
9.3t	RNA	AUGGGGACUAUACCGCGUAAUGCUGCCUCCCCAU - idT		*In vitro*		(130)
16.3	RNA	UACAGAGGAGUACAAGUAGCAUGAUCCCUCGUGUAAA	FX	*In vitro*	Block the initiation of coagulation	(55)
RNA11F7t	RNA	GAGAGCCCCAGCGAGAUAAUACUUGGCCCCGCUCUU	FXa	*Ex vivo*	Inhibit thrombin formation	(57)
RNABA4	RNA	GGUCGAUCACACAGUUCAAACGUAAUAAGCCAAUGUACGAGGCAGACGACAAAGCCCCAGCGAGAUAAUACUUGGCCCCGCU	FXA + FIIa	*In vitro*	Reduce clot formation	(58)
T18.3	RNA	GGACUUGGAUAACCUCACCGCAAUGGCGGCUUGUCAGACGACUCGCUGAGGAUCCGAG	FV	*In vitro*	Reduce clot formation	(56)
HD1	DNA	GGTTGGTGTGGTTGG	FIIa (Thrombin)	*In vivo*	Inhibit clot formation	(61)
NU172	DNA	CGCCTAGGTTGGGTAGGGTGGTGGCG		Phase II		NCT00808964
M08s-1	DNA	AGGTCAGATGATGGGGATGGGGGGTTGGAGGAATGGATGACCT		*In vivo*		(14)
RE31	DNA	CACTGGTAGGTTGGTGTGGTTGGGGCCAGTG		*In vitro*		(131)
RA36	DNA	GGTTGGTGTGGTTGGTGGTTGGTGTGGTTGG		*In vitro*		(132)
R9D-14T	RNA	GGCGGUCGAUCACACAGUUCAAACGUAAUAAGCCAAUGUCGAGGCAGACGACUCGCC *2’-Fluoro nucleotides*		*In vitro*		(133)
TBA-F14	DNA	GGTTGGTGTGGTTGG 8-trifluoromethyl-2′-deoxyguanosine		*In vitro*		(134)
CTBA4T-B1	DNA	TAGGGGGCGCGAACATACGCGGTTGGTGTGGTTGGCTGACTCGTATCTCGAGTCA		*In vitro*		(135)
HD22	DNA	AGTCCGTGGTAGGGCAGGTT GGGGTGACT		*Ex vivo*		(64)
Tog25	RNA	GAACAAAGCUGAAGUACUUACCCAAGAUCAUCCCGAACGA		*In vitro*		(65)
HD1-HD22	DNA	GGTTGGTGTGGTTGGAAAAAAAAAAAAAAAAGTCCGTGGTAGGGCAGGTTGGGGTGACT		*In vitro*		(66)
M08s-1-HD22	DNA	AGGTCAGATGATGGGGATGGGGGGTTGGAGGAATGGATGACCTTTTTTTTTTTTTTTTAGTCCGTGGTAGGGCAGGTTGGGGTGACT		*In vivo*		(14)
FA12	DNA	AAGCAGTGGTAAGTAGGTTGATCATCCTCTTTAAGTCATCACTTTAGTTTCTCCATCTACATCTCTTCGAGCAATCCACAC	FXIIIa	*In vitro*	Reduce clot rigidity and can allow fibrinolysis	(76)
AT-16	RNA	*Not referenced*	Antithrombin	*In vitro*	Enhance endogenous anticoagulant properties of antithrombin	(70)
R10-4	RNA	CCAGGCGUCUCACUCGUUACGCUAUCGUUGCGUACUUCUG	PAI-1	*In vitro*	inhibit PAI-1's antiproteolytic activity against tPA	(74)
R10-2	RNA	CACACGAGGCAAGUGGCCUGCAUAACGUAGGCGUCGAGUA				
KU7	RNA	GGGAGGACGAUGCGGACUGGUGAAGGGAGGUACUGCAGACGACUCGCCCGA	Histones H3 and H4	*In vivo*	Inhibit histone-induced platelet aggregation	(80)
NX21909	DNA	AGGACGATGCGGCACGGTAGTGCTACCAGATGGTTATGTTAC + Splint oligo: valyl phosphonate - GGTGCCGCATCGTCCT	Neutrophil elastase	*In vivo*	Reduce the recruitment and influx of neutrophils	(81)
CG51	DNA	CAACGTGTGATATGTGGGTATACGCTTGGGTGTTACGCTGAGCACAGAGGGTATTCGTGT	Cathepsin G	*In vitro*	Modulate proteolytic activity of cathepsin G	(82)
SL1102	DNA	CGC-2NEdU-GAGAA-2NEdU-AGAAG-2NEdU-AGGAG-2NEdU-A-2NEdU-GC-2NEdU-2NEdU-GCG 2-O-Methyl nucleotides	Factor B	*In vitro*	Regulate the alternative pathway’s activity	(86)
SL1103	DNA	GC-2NEdU-GAGAA-2NEdU-AGAAG-2NEdU-AGGAG-2NEdU-A-2NEdU-GC-2NEdU-2NEdU-GC 2-O-Methyl nucleotides				
Izervay™	RNA	43 kDa PEG -CGCCGCGGUCUCAGG CGCUGAGUCUGAGUUUACCUGCG-idT 2-O-Methyl nucleotides *2’-Fluoro nucleotides*	C5	Approved	Prevent C5 cleavage and decrease membrane attack complex formation	NCT04435366
NOX-D20	RNA	40 kDa PEG – GCGAUGUGGUGGU GAAGGGUUGUUGGGUGUCGACGCACGC Deoxyribonucleotides	C5a	*In vivo*	Attenuate inflammation and organ damage	(94)

idT: inverted T nucleotides, NH: hexylamine linker, T’: biotin-dT, Ds: 7-(2-thienyl)imidazo[4,5-b]pyridine, 2NEdU: 5-[N-(2-naphthylethylcarboxyamide)-2′-deoxyuridine replace T nucleotides.

**Table 3:** Summary table of aptamers with potential diagnostic applications for sepsis-induced coagulopathy.

Name	Type	Target	Diagnosis / Prognosis tools	Limit of detection (LOD) or Affinity (K_D_)	Reference
NOX-S93	RNA	Sphingosine-1-phosphate	Level inversely related to endothelial glycocalyx degradation	K_d_ = 4.3 nM	(104)
11-1.41	RNA	Angiopoietin 2	Level related to increase of vascular permeability	K_d_ = 2.2 nM	(106)
M55	DNA	Thrombospodin-1	Level related to endothelial dysfunction	LOD = 6.96 fM	(110)
Anti-kinin B1 receptor aptamer	RNA	Kinin B1 receptor	Level related to hypotension and increased vascular permeability	Affinity not determined	(113)
Anti-human neutrophil elastase aptamer	DNA	Neutrophil elastase	Level related to endothelial damage and coagulation cascade activation	LOD = 20 fM	(114)
Anti-PCT aptamer	DNA	PCT	Level related to bacterial infections	LOD = 0.01 ng/mL	(97)
CD63-1	DNA	CD63	Levels related to platelets in an active state	K_d_ = 38.71 nM	(99)
AT-1	DNA	CD31		K_d_ = 2.28 nM	(100)
iodo-FA	DNA	Fibrinogen	Level related to coagulation disorder	K_d_ = 9.75 μM	(136)
HS02-88	DNA	APC	Level inversely related to prothrombotic state	K_d_ = 0.43 nM	(117)
BAX499 / ARC19499	RNA	TFPI	Level inversely related to procoagulant effects	K_d_ = 2.8 nM	(119,120)
Aptamer 3218	RNA	tPA	Circulate tPA or uPA levels related to mortality	K_d_ ≈ nM	(122)
upanap-126	RNA	uPA		Affinity not determined	(123)
WT-15	DNA	PAI-1	Level related to thrombotic disorders	K_d_ = 177 pM	(125)

If referenced, the dissociation constant is given for aptamers that are not yet developed as sensor tools.

## Targeting the endothelial permeability and dysfunction

Inflammatory stimuli in sepsis induce a pro-inflammatory, procoagulant, and proadhesive phenotype in endothelial cells. Under physiological conditions, the endothelium plays a crucial role in vascular homeostasis by expressing antithrombotic and anti-inflammatory mediators such as nitric oxide, prostacyclin (PGI₂), thrombomodulin, and ADAMTS13. Its surface glycocalyx, a negatively charged carbohydrate-rich layer, further contributes to vascular protection by maintaining barrier integrity, responding to shear stress, and preventing leukocyte and platelet adhesion as well as activation of the coagulation cascade [19].

In sepsis, however, the endothelium is directly activated by pro-inflammatory cytokines (e.g., TNF-α, IL-6, IL-1), PAMPs, and indirectly by NETs. This leads to degradation of the glycocalyx and increased endothelial permeability due to exposure of the underlying membrane [19].

### Protecting the endothelial glycocalyx with aptamers

**Fig. 3:**
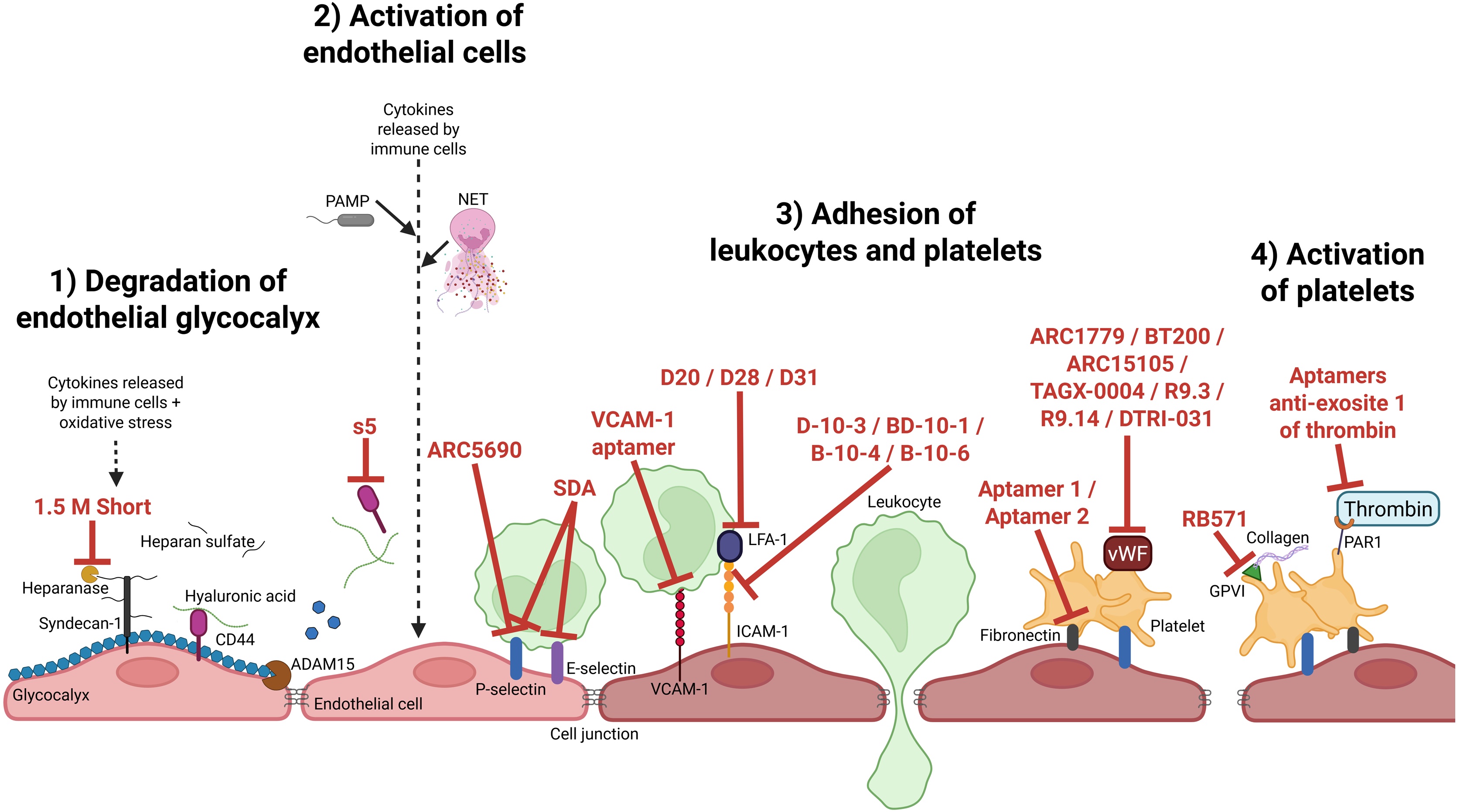
**Aptamers targeting endothelial dysfunction in septic DIC.** Glycocalyx damage (1) induced vascular permeability and expose endothelium to cytokines, PAMP and immune cells which will activate endothelial cells (2). Afterwards leukocytes and platelets adhere to endothelium (3) and platelets are activated (4). Aptamers targeting these different systems are shown in red. NET: neutrophil extracellular trap, PAMP: pathogen associated molecular pattern, LFA-1: lymphocyte function-associated antigen 1, ICAM-1: intercellular adhesion molecule 1, VCAM-1: vascular cell adhesion molecule 1, vWF: von Willebrand factor, GPVI: glycoprotein VI, PAR: protease-activated receptor.

One of the major challenges in sepsis is to protect and/or restore endothelial function to preserve vascular integrity and limit downstream complications. Glycocalyx degradation is mainly mediated by heparanase, which cleaves heparan sulfate, and ADAM15, which targets CD44. We hypothesize that aptamers directed against these enzymes or their binding sites could preserve glycocalyx integrity. For example, anti-heparanase aptamers developed in oncology have been shown to inhibit tumor cell invasion in *in vitro* carcinoma models [20]. Similarly, a DNA aptamer targeting the hyaluronic acid-binding domain of CD44 (aptamer s5, K_D_ = 779 nM) inhibited proliferation of leukemia cell lines (*in vitro*), suggesting possible utility in preventing CD44 cleavage by ADAM15 [21] (Fig. 3).

### Targeting endothelial junctions and barrier function

Endothelial glycocalyx disruption leads to exposure of the endothelium and loss of tight and *adherens* junction integrity. Elevated serum lactate—associated with sepsis severity—disrupts vascular endothelial cadherin, leading to gap formation between endothelial cells and vascular leakage [22]. Inflammatory cytokines or NF-κB pathway activation also reduce the expression of key tight junction proteins (e.g., occludin, ZO-1, claudin-5), facilitating leukocyte transmigration [23]. To date, no aptamers have been developed to directly target endothelial junction proteins such as VE-cadherin, occludin, claudin-5, or ZO-1, but this might represent a potential area for future research aimed at preserving vascular integrity in sepsis.

### Aptamers against endothelial activation and leukocyte adhesion

Activated endothelial cells release DAMPs that amplify inflammation and undergo phenotypic changes, including oxidative stress and increased expression of adhesion molecules such as selectins, integrins, ICAM-1, and VCAM-1. These molecules facilitate leukocyte rolling, adhesion, and extravasation. Their circulating levels (ICAM-1, VCAM-1, E-selectin, P-selectin, and vWF) correlate with sepsis severity and poor outcomes [24,25].

Aptamers targeting adhesion molecules represent promising therapeutic tools to block endothelial-leukocyte interactions, limit barrier disruption, and modulate inflammatory signaling. Several aptamers targeting ICAM-1, VCAM-1, LFA-1, as well as P- and E-selectins, have demonstrated functional effects both *in vitro* and in mouse models of transient cerebral ischemia miming a stoke or sickle cell disease [[26], [27], [28], [29], [30]].

These examples highlight the potential of aptamers to modulate key events in sepsis-induced endothelial dysfunction, thereby reducing vascular leakage, inflammation, and possibly downstream coagulopathy (Fig. 3).

Building on this, another critical therapeutic target in sepsis-induced coagulopathy is the excessive activation of the coagulation cascade, which contributes to microthrombus formation and organ dysfunction.

## Targeting dysregulated coagulation pathways

During sepsis, endothelial glycocalyx degradation exposes procoagulant surfaces and increases vascular permeability. Activated or damaged endothelial cells release von Willebrand factor (vWF), promoting platelet adhesion and aggregation. Activated platelets expose phosphatidylserine, which amplifies the intrinsic coagulation pathway. This pathway is also triggered by microvesicles from platelets, immune, and endothelial cells. In parallel, inflammatory stimuli upregulate tissue factor (TF) on immune and endothelial cells, activating the extrinsic pathway and leading to excessive thrombin generation [31]. Elevated thrombin further promotes platelet activation and fibrin formation, contributing to microthrombi.

This hypercoagulable state overwhelms endogenous anticoagulant systems. Antithrombin (AT) and thrombomodulin (TM) levels decrease, resulting in reduced activated protein C (APC), while tissue factor pathway inhibitor (TFPI) becomes insufficient to counteract the procoagulant cascade.

In this context, aptamers could offer a therapeutic strategy by blocking platelet adhesion, inhibiting platelet or coagulation factor activation, or enhancing anticoagulant pathways.

### Reducing platelet aggregation and adhesion

During haemostasis, platelets adhere and aggregate at sites of endothelial injury to maintain vascular integrity and prevent bleeding. This process involves adhesion to exposed subendothelial components such as vWF, collagen, fibronectin, and fibrinogen. A potential strategy involves using DNA aptamers targeting *in vitro* fibronectin to inhibit fibronectin–integrin-mediated adhesion (Fig. 3) [32].

In sepsis and DIC, elevated vWF antigen levels are markers of endothelial activation and disease severity [33]. vWF is normally cleaved by ADAMTS13 into less thrombogenic fragments. However, in sepsis, excessive vWF release consumes ADAMTS13, resulting in a prothrombotic imbalance. Several aptamers targeting vWF have been developed (Fig. 3). ARC1779 binds the A1 domain of activated vWF, blocking its interaction with the GPIb receptor on platelets. It has shown good tolerability with no bleeding complications in a first-in-human trial (NCT00432770) [34]. ARC1779 inhibits all vWF-mediated platelet activation pathways and has been evaluated in five phase II trials (NCT00632242, NCT00726544, NCT00694785, NCT00507338, NCT00742612) for conditions such as thrombotic thrombocytopenic purpura [35,36], von Willebrand disease type 2B [37], acute myocardial infarction [38], and cerebral thromboembolism after carotid endarterectomy [39].

Other vWF-targeting aptamers including BT200, ARC15105, TAGX-0004, R9.3, R9.14, and DTRI-031 also reduce platelet adhesion and clot formation in *in vitro, ex vivo* and *in vivo* models (Figure 3) [[40], [41], [42], [43], [44]]. BT200 reduced platelet adhesion and thrombus formation in *ex vivo* models using healthy volunteers (stimulated with desmopressin or LPS). In parallel, the aptamer DTRI-031 effectively dissolves platelet-rich clots in carotid artery (mouse and canine) and saphenous vein (mouse) thrombosis models, underscoring its potential therapeutic value for thrombosis management in non-pathological settings. These findings highlight the potential of aptamers to disrupt vWF–GPIb-V-IX interactions and limit platelet adhesion.

Moreover, P-selectin aptamers (discussed in Part 2) could inhibit platelet–endothelium adhesion by targeting P-selectin/PSGL-1 interactions (Fig. 3), potentially reducing microthrombus formation in sepsis, although the degree of protection may vary depending on the experimental model and underlying inflammatory context.

In addition to adhesion, platelet activation plays a key role in thrombo-inflammation. Platelets are activated by pathogens via PRRs, GPVI, and FcγRIIA, and by soluble agonists like thrombin (via PAR1/PAR4), ADP (via P2Y1/P2Y12), and thromboxane A₂ (via TP receptor). Activated platelets release granules with prothrombotic and pro-inflammatory contents and shed microvesicles bearing TF and phosphatidylserine.

Aptamers targeting platelet activation pathways may reduce this procoagulant activity. The RB571 aptamer targets GPVI and inhibits collagen-related peptide-induced platelet aggregation, with an IC_50_ of 30 nM *in vitro* [45] (Fig. 3). Aptamers targeting thrombin exosite I have also been shown to block thrombin–PAR1 interaction and platelet activation [46]. Since activated platelets provide a phosphatidylserine-rich surface for intrinsic coagulation activation, aptamers interfering with platelet activation may offer a targeted anticoagulant effect.

Together, these approaches suggest that aptamers may improve therapeutic outcomes in sepsis-associated coagulopathy by limiting platelet adhesion, activation, and potentially downstream thrombus formation.

### Inhibiting factors of the coagulation cascade

A stable blood clot is formed during the second phase of haemostasis through a series of enzyme-driven reactions that stabilize the initial platelet plug. The coagulation cascade is divided into three interconnected pathways: intrinsic, extrinsic, and common.

#### Intrinsic pathway: FXII and FXI inhibition

Activation of factor XII (FXII) initiates the intrinsic pathway and proceeds via FXI and FIX, ultimately activating FX. Exposed phosphatidylserines on microvesicles interact with FXII, inducing conformational changes that promote its autoactivation (contact activation). This process is enhanced by collagen, kininogen, and proteolytic enzymes such as kallikrein, thrombin, and trypsin. As discussed above, endothelial activation in sepsis promotes a procoagulant and proinflammatory state, contributing to microvascular thrombosis.

Importantly, the FXII/FXI pathway contributes to thrombus growth but is dispensable for physiological haemostasis [47], making it an attractive therapeutic target. The aptamer R4cXII-1 inhibits, *in vitro*, FXII autoactivation and downstream FXI activation, delaying clot formation. However, it does not block FXIIa activation mediated by the kallikrein–kinin system [48].

**Fig. 4:**
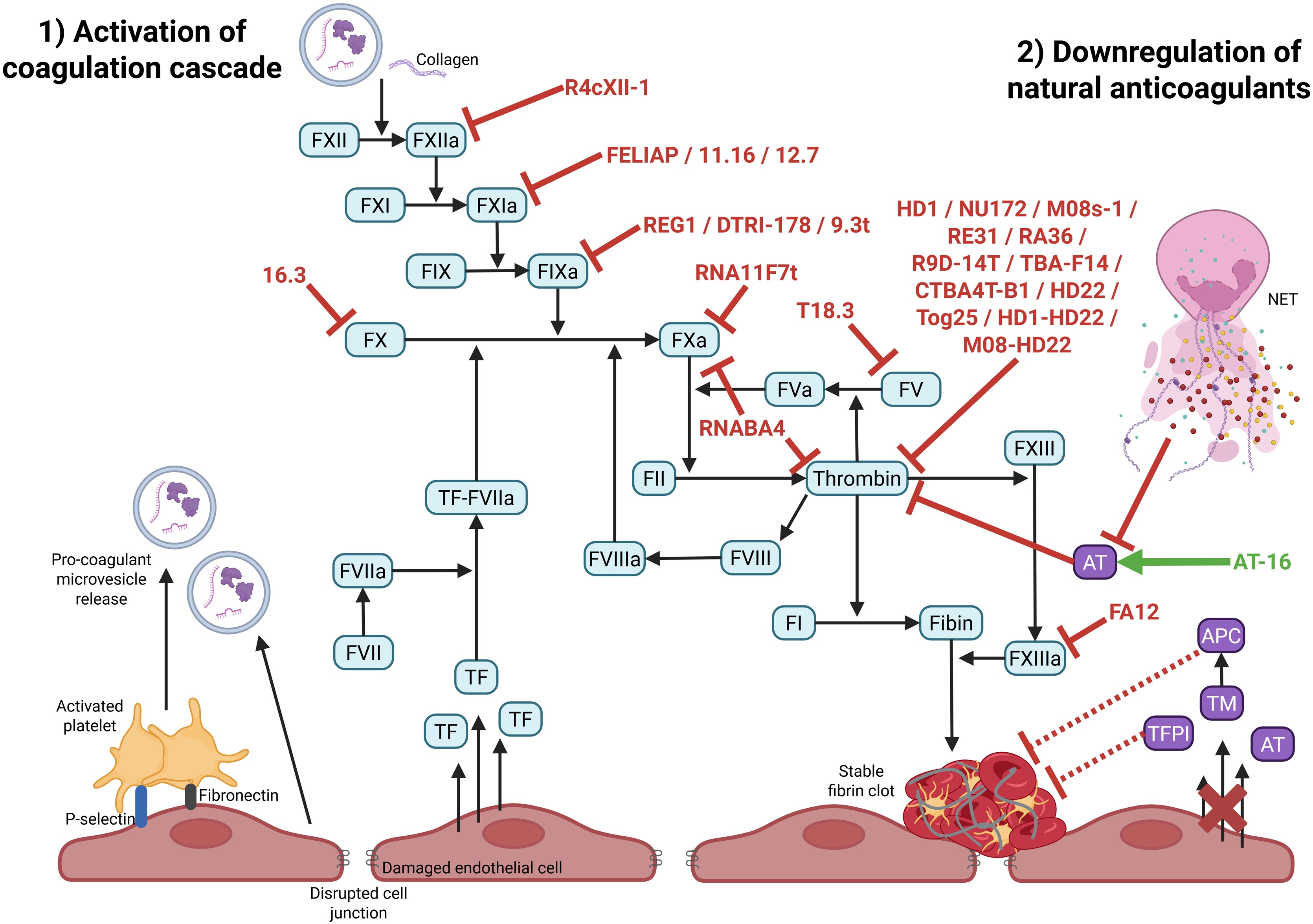
**Aptamers targeting excessive coagulation activation.** Excess of coagulation activation (1) and downregulation of endogenous anticoagulants (2) in septic DIC forms stable fibrin clot responsible of organ failure. Inhibitor aptamers are in red whereas agonist aptamer is in green. The coagulation cascade factors are highlighted in cyan. Endogenous anticoagulants are represented in purple. NET: neutrophil extracellular trap, TF: tissue factor, FXIIa: Factor XII activated, AT: antithrombin, TM: thrombomodulin, APC: activated protein C, TFPI: tissue factor pathway inhibitor.

In addition, several aptamers have been developed to target *in vitro* FXIa, including the Factor Eleven Inhibitory Aptamer (FELIAP) [49], and the 11.16 and 12.7 aptamers [50]. These molecules prolong clotting times by approximately 1.3- to 2-fold through inhibition of the intrinsic coagulation pathway, which suppresses thrombin generation and consequently delays clot formation (Fig. 4). Inhibition of FXIa is particularly attractive as it reduces thrombosis while exerting only a limited impact on physiological haemostasis, thereby lowering the risk of bleeding compared to conventional anticoagulants.

#### FIXa inhibition: the REG1 system and others

The REG1 system comprises RB006, an RNA aptamer targeting FIXa, and its complementary RNA antidote RB007, allowing precise and reversible anticoagulation. REG1 demonstrated efficacy and safety in phase II clinical trials for acute coronary syndrome (NCT00932100) and coronary artery disease (NCT00715455) [51]. However, phase III development was halted due to severe allergic reactions, likely related to PEG conjugated to the 3' end of RB006 [52].

Despite this, the antidote RB007 showed strong efficacy in restoring clotting times to baseline levels, highlighting the therapeutic potential of aptamer-antidote systems [53]. Additional FIXa-targeting aptamers have been identified. Among these, DTRI-178 was evaluated in a veno-arterial extracorporeal membrane oxygenation (ECMO) model in pigs. This study assessed ECMO-induced coagulopathy and showed that a single dose of aptamer effectively prevented thrombosis. Its anticoagulant efficacy was comparable to that achieved with continuous unfractionated heparin (UFH) infusion. Notably, DTRI-178 was associated with reduced bleeding at the surgical site and a lower transfusion requirement compared with UFH [54]. Overall, these aptamers demonstrated effective anticoagulant activity and expanded the range of FIXa-targeted therapeutic options (Fig. 4).

#### Extrinsic pathway: TF–FVIIa complex

The extrinsic pathway is activated by TF exposure, typically from damaged tissues. In sepsis, TF is also highly expressed by immune cells, activated endothelium, and platelets, as well as their released microvesicles, contributing to upregulation of the extrinsic coagulation pathway.

The RNA aptamer 16.3 blocks *in vitro* the formation of the TF–FVIIa complex, thereby preventing FX activation and prolonging prothrombin time by 1.8-fold in human plasma [55]. With a plasma half-life of 15 h *in vitro*, the aptamer 16.3 is a promising candidate for *in vivo* use in sepsis-induced coagulopathy, although further studies are needed to establish its pharmacokinetic profile and therapeutic efficacy under physiological and pathological conditions (Fig. 4).

#### Common pathway: FX, FXa/FVa complex, and thrombin

FX is activated by both the intrinsic (FIXa, with FVIIIa) and extrinsic (TF–FVIIa) pathways. FXa then forms a complex with FVa to convert prothrombin (FII) into thrombin (FIIa), which cleaves fibrinogen into fibrin and activates FXIII to stabilize the clot.

Disrupting the FXa–FVa complex could inhibit thrombin generation independently of upstream pathways. The aptamer T18.3 binds both FV and FVa, displaying anticoagulant activity in plasma from COVID-19 patients [56]. Another strategy is to directly inhibit FX or FXa using RNA11F7t, which prevents activation and interaction with FVIIIa [57]. It was studied *ex vivo* using healthy human blood circulated through an extracorporeal oxygenator circuit designed to reproduce cardiopulmonary bypass conditions. In this setting, RNA11F7t was used to investigate coagulopathy related to extracorporeal circulation in a surgical, non-infectious context. To further improve efficacy, multimeric aptamers have been developed. The bivalent aptamer RNABA4 combines RNA11F7t and R9D-14T, simultaneously targeting FX/FXa and thrombin. This dual mechanism inhibits *in vitro* both thrombin generation and fibrin formation, amplifying anticoagulant effects and providing precise control of coagulation [58].

#### Thrombin inhibition: exosite I, exosite II, and dual targeting

Thrombin, a central enzyme in coagulation, has two exosites: exosite I, which binds fibrinogen, thrombin receptor, and thrombomodulin, and exosite II, which binds heparin and prothrombin domains [59].

The first thrombin-binding aptamer, HD1 (also known as ARC183 or TBA), was discovered in 1992 [60]. It was studied in the cynomolgus monkey in an extracorporeal circulation model [61]. However, its clinical development was discontinued due to high dose requirements [62]. Subsequently, several next-generation aptamers have been designed to target thrombin exosite I with greater affinity and specificity, although most of them have been evaluated only in vitro [[71], [72], [73], [74], [75]]. The aptamer NU172 has been tested in a phase II clinical trial for off-pump coronary artery bypass grafting (NCT00808964) [63]. Another anti-exosite I aptamer, M08 has been prolonging clotting time in healthy mice [63].

Exosite II–binding aptamers, though generally less potent, are valuable for more selective modulation. HD22, one of the earliest, binds exosite II and inhibits activation of coagulation cofactors V and VIII, interfering with clot amplification [64]. HD22 has been studied *ex vivo* with human blood physiological flow conditions. Other aptamers such as Tog25 also inhibit *in vitro* clot formation and platelet activation [65] (Fig. 4).

Bivalent aptamers that simultaneously bind exosites I and II show enhanced anticoagulant effects. HD1-HD22 (*in vitro*) and M08-HD22 (in healthy mice) combine the specificity of HD1 or M08 with HD22 [66,67]. Optimizing linker length and composition produced a synergistic effect, with efficacy up to four times that of individual aptamers [67]. These aptamers disrupt both initiation and amplification phases of thrombin-mediated coagulation, reducing thrombus formation while potentially minimizing bleeding risk.

### Counteracting downregulation of endogenous anticoagulants

In sepsis-induced DIC, endogenous anticoagulant systems are significantly downregulated, contributing to the procoagulant state characteristic of DIC. The main anticoagulants affected include antithrombin (AT), thrombomodulin (TM), and tissue factor pathway inhibitor (TFPI).

TM, an endothelial membrane receptor, forms a complex with thrombin to activate protein C (in the presence of protein S), which then inactivates FVa and FVIIIa. In sepsis, endothelial dysfunction leads to the shedding of TM, increasing soluble TM levels in plasma and reducing its availability on the cell surface, resulting in insufficient generation of activated protein C (APC). Moreover, TM downregulation contributes to hyperinflammation, as HMGB1, a key inflammatory mediator usually cleaved and inactivated by the thrombin–TM complex, remains active in septic DIC.

In parallel, TFPI is consumed through its binding to FXa and the TF–FVIIa complex. Inflammatory stimuli also suppress TFPI synthesis in endothelial cells and monocytes. Despite these insights, replacement therapies using exogenous AT, APC, or TM have not improved patient outcomes, and often increased bleeding risks [68].

An alternative strategy would be to counteract the downregulation of these endogenous anticoagulants by enhancing their function or availability, rather than supplementing them directly. This could restore physiological anticoagulant balance without increasing bleeding risk, a key limitation of conventional therapies.

AT is a serine protease inhibitor that neutralizes key coagulation enzymes such as thrombin and FXa. During sepsis, AT levels decrease due to: [1] consumption through formation of irreversible complexes with thrombin and FXa [2], loss of heparan sulfate from the endothelial surface, which is required to activate AT, and [3] degradation by neutrophil elastase during NETosis [69].

Although aptamers are typically used to inhibit targets, they can also act as agonists, binding their targets to induce beneficial conformational changes. AT-16 is an RNA aptamer that specifically binds AT and enhances *in vitro* its anticoagulant activity by increasing its affinity for FXa (Fig. 4) [70]. By promoting this conformational shift, AT-16 potentiates AT’s inhibition of FXa, reducing thrombin generation and subsequent clot formation.

Unlike heparin, which also activates AT but binds multiple targets (e.g., heparin cofactor II, protein C inhibitor, TFPI), AT-16 exhibits higher specificity and affinity for AT, potentially reducing off-target effects [71]. While the efficacy and safety of the aptamer-mediated enhancement of AT activity in sepsis-induced DIC, particularly regarding bleeding risk, still require confirmation in clinically relevant models, this approach holds promise as a novel strategy to restore anticoagulant balance in this condition.

## Restoring defective fibrinolysis in sepsis-induced DIC

Fibrinolysis and coagulation tightly interact to maintain haemostatic balance. The primary role of fibrinolysis is to degrade fibrin networks within clots. Endothelial cells release tissue plasminogen activator (tPA), which converts plasminogen into plasmin, the main enzyme responsible for clot dissolution. In patients with sepsis-induced DIC, fibrinolysis is impaired [72], mainly due to elevated levels of plasminogen activator inhibitor-1 (PAI-1), which blocks both tPA and urokinase-type plasminogen activator (uPA), preventing plasminogen activation and promoting a prothrombotic state [73].

**Fig. 5:**
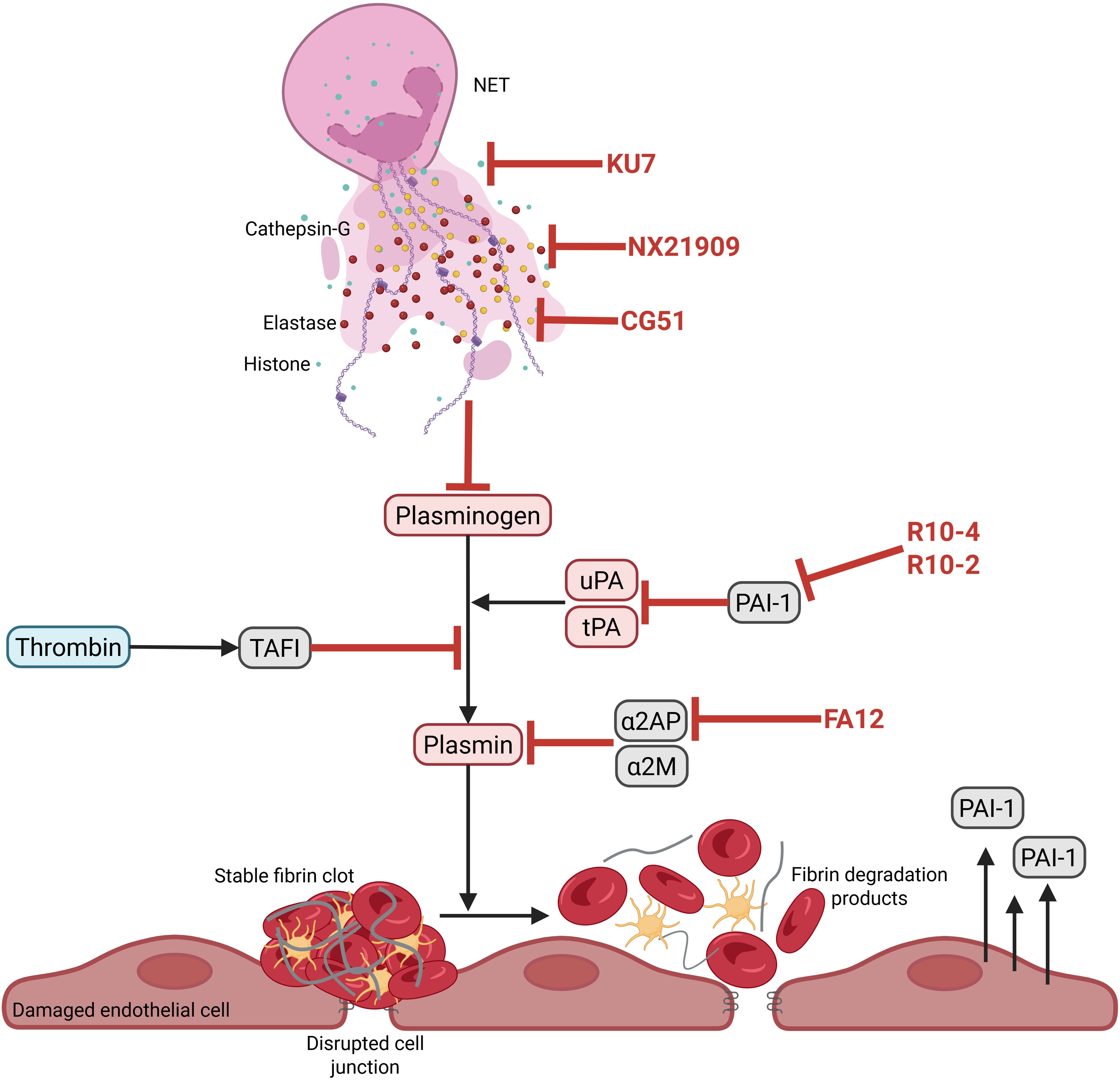
**Aptamers targeting the fibrinolytic pathway in septic DIC.** Aptamers are in red. Fibrinolytic system is shown in coral-colored. Fibrinolysis inhibitors are in grey. Thrombin refers to coagulation pathway occurs in cyan. NET: neutrophil extracellular trap, uPA: urokinase plasminogen activator, tPA: tissue-type plasminogen activator, PAI-1: plasminogen activator inhibitor type-1, α2AP: α2-antiplasmin, α2M: α2-macroglobulin, TAFI: thrombin activatable fibrinolysis inhibitor.

To counteract this, aptamers targeting PAI-1 have been developed. Among them, R10-4 and R10-2 specifically inhibit *in vitro* the anti-proteolytic activity of PAI-1 against tPA, thereby enhancing fibrinolysis by allowing tPA to more effectively convert plasminogen to plasmin [74] (Fig. 5). These aptamers represent a promising therapeutic strategy in settings where excessive PAI-1 suppresses fibrinolysis. However, their clinical efficacy and safety remain to be demonstrated *in vivo*, considering the intricate regulation of fibrinolysis and potential off-target effects in septic coagulopathy. In addition to excessive PAI-1, free plasmin that does form in septic DIC is rapidly neutralized by α2-antiplasmin [75]. This leads to the formation of plasmin–antiplasmin complexes, further impairing fibrinolysis. Moreover, FXIIIa, activated by thrombin, stabilizes clots by crosslinking fibrin and incorporates α2-antiplasmin into the fibrin network, protecting it from degradation.

Inhibiting FXIIIa could thus help restore fibrinolysis. *In vitro* studies showed that aptamers targeting FXIIIa reduce clot formation. Notably, the FA12 aptamer inhibits FXIIIa-mediated incorporation of α2-antiplasmin into fibrin, reducing clot rigidity and increasing susceptibility to lysis [76] (Fig. 5).

Other inhibitors of fibrinolysis include thrombin-activatable fibrinolysis inhibitor (TAFI) and α2-macroglobulin. TAFI, once activated by thrombin, is strongly potentiated (∼1250-fold) by thrombomodulin [77]. Active TAFI cleaves C-terminal lysine residues from fibrin degradation products, removing key binding sites for tPA and plasminogen. This disrupts the positive feedback loop that enhances plasmin generation.

Finally, α2-macroglobulin serves as a secondary inhibitor of plasmin when α2-antiplasmin is depleted. Thus, developing aptamers targeting TAFI, α2-antiplasmin, or α2-macroglobulin could represent promising future strategies to restore fibrinolysis in sepsis-induced coagulopathy.

## Targeting neutrophil-derived mediators and NETs to decrease excessive coagulation activation and restore fibrinolysis

Neutrophils play a central role in innate immunity by migrating to sites of infection or injury and exhibiting antimicrobial functions. Their release into peripheral blood is tightly regulated by the CXCR4–CXCL12 axis, but during sepsis, CXCL12 is downregulated, leading to increased neutrophil release [78]. In circulation, neutrophils become more deformable and express integrins that enable margination and adhesion to the endothelium via selectins. Bacterial components in sepsis enhance expression of β1/β2 integrins on neutrophils and upregulate ICAM-1 and VCAM-1 on endothelial cells, promoting firm adhesion. Transmigration to infected tissues is mediated by chemokine–CXCR2 interactions, though CXCR2 is progressively downregulated in later stages of sepsis [79].

Neutrophils release both pro- and anti-inflammatory mediators and are therefore potential therapeutic targets to mitigate excessive coagulation and restore fibrinolysis in sepsis-induced coagulopathy.

One major neutrophil effector mechanism is NETosis, a specialized form of programmed cell death triggered by PAMPs, cytokines, and activated platelets. During NETosis, neutrophil elastase and myeloperoxidase translocate to the nucleus, leading to chromatin decondensation and rupture of the nuclear envelope. The resulting NETs are composed of DNA, histones (H3, H4), and granule proteins (elastase, cathepsin-G, MPO), and function to immobilize and kill pathogens.

While beneficial in infection control, excessive NET formation and impaired clearance contribute to endothelial damage and thrombo-inflammation. During sepsis, NETs adhere to and activate endothelial cells via histone-dependent mechanisms. Histones stimulate TLR pathways, promoting cytokine release and vascular injury.

RNA aptamers targeting histones H3 and H4 have been shown to neutralize their proinflammatory activity in human cells and in a murine model of multiple organ dysfunction induced by intravenous injection of calf thymus histones (Figure 5) [80]. These aptamers may reduce cytokine release, endothelial activation, and NET-associated damage.

NETs also act as a scaffold for platelet adhesion and aggregation, and their DNA/protein content activates FXII, promoting fibrin generation. Moreover, neutrophil elastase, a key NET component, degrades plasminogen, limiting plasmin generation and impairing fibrinolysis [9].

Aptamers targeting neutrophil elastase may help restore fibrinolysis. The RNA aptamer NX21909 inhibits elastase activity in a rat model of acute lung injury induced by tracheal administration of IgG anti-bovine serum albumin (BSA) then BSA intravenous injection, reducing both tissue damage and neutrophil recruitment to the lungs [81] (Fig. 5). By limiting plasminogen degradation and inflammatory infiltration, such aptamers could counteract neutrophil-driven fibrinolytic failure in sepsis.

Another NET-associated protease, cathepsin-G, contributes to extracellular matrix degradation, platelet aggregation, and proinflammatory signaling. While essential in host defense, excess cathepsin-G activity aggravates tissue injury in inflammatory conditions like sepsis. The CG51 aptamer, developed to inhibit cathepsin-G, reduces *in vitro* its proteolytic activity and may help rebalance immune responses while limiting tissue destruction [82].

These aptamers demonstrate the potential to selectively modulate NET components, reduce thrombo-inflammation, and mitigate endothelial injury. Nevertheless, their efficacy in fully restoring fibrinolysis and preserving endothelial integrity in septic coagulopathy remains to be validated in clinical studies, given the complexity of NET-mediated pathophysiology. Building on these mechanisms, another critical driver of sepsis-induced DIC is the complement system, which tightly interconnects with coagulation and inflammation.

## Targeting complement system in sepsis-induced DIC

The complement system and coagulation cascade are closely interconnected. Complement proteins can activate coagulation, while coagulation factors such as thrombin and FXa can directly activate the complement system, establishing a positive feedback loop that contributes to the development of DIC [83]. This process is exacerbated by the downregulation of anticoagulants like thrombomodulin (TM) and activated protein C (APC), which normally inhibit complement activation [84]. Loss of these regulatory mechanisms amplifies both coagulation and inflammation, worsening the clinical course.

Given this interplay, the complement system represents a promising therapeutic target in sepsis-induced DIC. A randomized controlled trial involving C1-inhibitor in 40 patients with severe sepsis or septic shock showed mild benefit in reducing organ dysfunction, likely due to broad inhibition of all three complement pathways [85].

At the center of complement activation is C3, which integrates signals from the classical, lectin, and alternative pathways. The classical pathway is triggered by antibodies and pentraxins (e.g., CRP, serum amyloid P, pentraxin 3), the lectin pathway by mannose-binding lectin recognizing pathogen-associated carbohydrates, and the alternative pathway by spontaneous C3 hydrolysis on foreign or damaged cells.

**Fig. 6:**
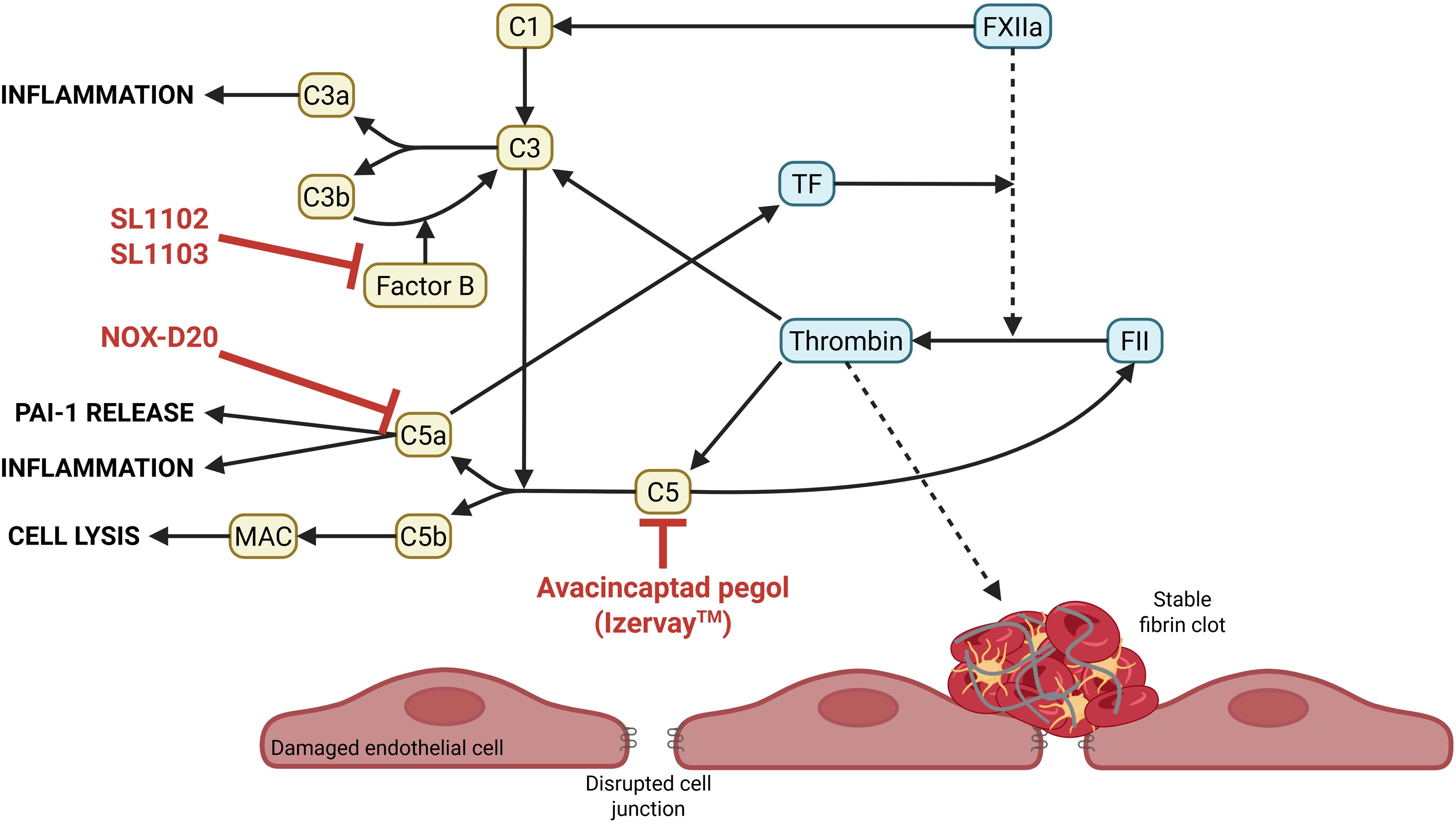
**Aptamers targeting the complement system exacerbating inflammatory and procoagulant states in septic DIC.** Aptamers are in red. Complement system is shown in yellow. Simplified coagulation pathway occurs in cyan. C1: complement 1, MAC: membrane attack complex, TF: tissue factor, FXIIa: Factor XII activated, PAI-1: plasminogen activator inhibitor type-1.

The alternative pathway is critical for amplifying C3 activation. Factor B is a key mediator that helps form C3 and C5 convertases, essential enzymes in the cascade. Several aptamers have been developed to specifically inhibit *in vitro* factor B, preventing C3 convertase formation and regulating the alternative pathway (Fig. 6) [86].

Upon activation, complement proteins are cleaved into two fragments: a smaller “a” subunit (anaphylatoxin) and a larger “b” subunit with binding activity. For instance, C3b amplifies its own production, opsonizes pathogens, and participates in C5 cleavage into C5a (a potent proinflammatory mediator) and C5b, which initiates the membrane attack complex (MAC). Excessive MAC formation can lead to tissue injury [87].

C3a and C5a, the main anaphylatoxins, recruit neutrophils and monocytes, increase vascular permeability, and upregulate endothelial adhesion molecules. Elevated levels of these fragments during sepsis have been linked to anaphylactic shock, endothelial damage, and coagulation activation, ultimately contributing to DIC [88,89]. Notably, C5a promotes procoagulant activity by upregulating tissue factor (TF) in endothelial cells and neutrophils, and by shifting mast cells and basophils from a profibrinolytic to a prothrombotic phenotype, via increased PAI-1 expression [[90], [91], [92]].

Aptamers targeting the complement system could offer new therapeutic opportunities (Fig. 6):

• Avacincaptad pegol (Iverzay™), an FDA-approved RNA aptamer (2023), blocks C5 cleavage into C5a and C5b. This inhibits both C5a-induced inflammation and MAC formation, offering protection from complement-mediated tissue damage. Although approved for geographic atrophy in age-related macular degeneration, its dual anti-inflammatory and cytoprotective action could be valuable in sepsis [93].
• NOX-D20, a Spiegelmer (L-nucleotide aptamer), binds C5a and competes with its receptor, thereby inhibiting C5a-induced immune responses. Due to its L-nucleotide structure, NOX-D20 is resistant to nuclease degradation, enhancing its stability *in vivo*. In a murine model of polymicrobial sepsis (cecal ligation and puncture), NOX-D20 reduced inflammation, prevented endothelial barrier breakdown, decreased organ injury, and improved the 7-day survival [94]. Survival did not differ between treatment groups or among the tested dosing regimens (two daily doses of 1 mg/kg and 3 mg/kg, or a single 1 mg/kg dose).

These findings support the therapeutic potential of complement-targeting aptamers in sepsis-induced DIC by simultaneously addressing inflammation, endothelial dysfunction, and coagulation activation. Nevertheless, their clinical efficacy and safety must be evaluated in patients with sepsis-induced coagulopathy to confirm their therapeutic potential.

## Aptamers for diagnosis and prognosis tools of sepsis-induced coagulopathy

In addition to their potential therapeutic applications in sepsis-induced DIC, aptamers may also be developed as diagnostic or prognostic tools (Table 3).

First, aptamers can help detect markers of infection. Alongside C-reactive protein, procalcitonin (PCT) is a well-established biomarker for early bacterial infections and is significantly elevated in patients with DIC [95,96]. An aptamer-based biosensor has been developed to detect PCT in clinical samples within 5 min, with a detection limit of 0.01 ng/mL [97]. Compared to conventional methods, this approach is faster, more sensitive, and requires smaller sample volumes.

Aptamers also offer potential in identifying DIC-related markers. Platelet count and fibrinogen are integral to the ISTH DIC score. In septic patients, platelets are often reduced and activated, expressing markers such as P-selectin, CD63, CD31, and soluble GPVI, and contributing to inflammatory aggregates [98]. Aptamers targeting CD63 and CD31 have been developed to isolate specific cell populations or exosomes [99,100]. Similarly, a fibrinogen-specific aptamer has shown high sensitivity in imaging thrombi, even at sub-nanomolar concentrations [101], suggesting utility for real-time thrombotic event monitoring [102].

Endothelial dysfunction can also be explored using aptamer tools. Sphingosine-1-phosphate (S1P), a component of the endothelial glycocalyx that preserves vascular integrity, is inversely associated with sepsis severity [103]. NOX-S93, an aptamer inhibiting S1P's proangiogenic activity, may serve as a prognostic marker [104]. Inflammatory signals in sepsis also induce angiopoietin-2 secretion, promoting glycocalyx degradation via endothelial heparanase [105]. RNA aptamers targeting angiopoietin-2 have been validated in angiogenesis models [106] and may be repurposed for sepsis. Similarly, thrombospondin-1, which modulates coagulation and is released during endothelial activation, has been detected with high sensitivity using aptamer-based colorimetric sensors [[107], [108], [109], [110]]. Finally, hypotension and increased vascular permeability induced by bacteria infection or endotoxemia are due to upregulation of the kinin B1 receptor in vascular tissues and increased bradykinin levels [111,112]. Aptamers targeting the kinin B1 receptor [113], or neutrophil elastase [114,115], may reflect vascular leakage and NETosis-related endothelial injury.

Aptamers could also be used to monitor anticoagulant levels. Protein C is reduced in septic DIC and serves as a prognostic marker [116]. The DNA aptamer HS02-88, initially developed to inhibit APC [117], may be repurposed to quantify APC levels in diagnostic assays. TFPI, another key anticoagulant downregulated in sepsis, is associated with disease severity [118]. While aptamers such as BAX499 and ARC19499 were developed as TFPI inhibitors for bleeding disorders [119,120], they demonstrate the feasibility of aptamer-based modulation and detection of TFPI.

Markers of hypofibrinolysis also deserve attention. tPA and uPA, activators of plasminogen, are inhibited in sepsis mainly by elevated PAI-1. Interestingly, the ProCESS trial found that high circulating tPA levels were associated with increased mortality [121], suggesting that tPA quantification could guide prognosis. The bivalent RNA aptamer 3218 binds tPA with high affinity and could be adapted for measurement [122]. Aptamers targeting uPA—originally developed to block tumor cell invasion [123]—may also have prognostic value, as uPA levels correlate with severity in septic shock [124]. Given PAI-1’s central role in suppressing fibrinolysis [73], aptamers such as R10-2 and R10-4 that block its inhibitory function [74,125] could serve dual roles as therapeutics and diagnostic tools, helping to stratify thrombotic risk in sepsis-induced DIC.

## Discussion

Nucleic acid aptamers were recognized as one of the top ten most promising emerging technologies in chemistry in November 2024 [126], and have appeared as a rapidly advancing class of molecules in clinical research. For therapeutic applications [127], 14 aptamers are currently undergoing clinical investigation across 23 Phase I and II trials, for different indications (cancer, cardiovascular disease, viral infections, autoimmune and inflammatory conditions), and two aptamers have received FDA approval: pegaptanib (Macugen®), approved in 2004 to inhibit VEGF in age-related macular degeneration [128]; and avacincaptad pegol (Izervay™), approved in 2023, to target the complement component C5, to treat geographic atrophy in age-related macular degeneration [93].

However, no aptamers have so far reached approval or advanced clinical stages for septic coagulopathy, and only one has been investigated in sepsis-related settings [94]. This limited progress is due to several factors. First, the complex and rapidly evolving pathophysiology of sepsis-induced coagulopathy complicates the development of appropriate preclinical models and hampers clinical translation, particularly because of patient stratification challenges. Other limitations are intrinsic to aptamer therapeutics. The major challenges for aptamer development are associated with their limited *in vivo* stability, suboptimal delivery efficiency, and intense market competition, particularly from monoclonal antibodies.

In this context, patients with sepsis-induced DIC may typically receive continuous intravenous treatments for supportive care. As a result, administration of an aptamer-based therapy through the same route would be both feasible and compatible with standard intensive care practices. For example, we have recently shown that 50% of anti-thrombin aptamers is conserved during 24 h in septic patient plasmas (unpublished results). Therefore, delivery route and short half-life are not expected to represent a limitation.

Aptamers also benefit from decades of accumulated knowledge in nucleic acid–based therapeutics, recently accelerated by the development of mRNA-based vaccines against SARS-CoV-2 during the COVID-19 pandemic, notably in the area of chemical stabilization. A high number of chemical modifications have been developed and validated for nucleic acids, enhancing *in vivo* stability, reducing immunogenicity, and improving pharmacokinetics. Advances in nucleic acid therapeutics can be directly applied to aptamer design, increasing the likelihood of developing clinically viable candidates. The diversity of targets and therapeutic potential highlighted in this review indicate that the 55 aptamers described are strong candidates for future clinical application in this field. Consequently, aptamer-based therapies hold considerable promise in the era of precision medicine, offering highly specific and tunable tools to modulate key components of inflammation, endothelial dysfunction, coagulation, and fibrinolysis. In the complex pathophysiology of sepsis-induced coagulopathy, aptamers provide the opportunity to selectively inhibit or enhance molecular targets involved in thrombosis and vascular injury, while minimizing the risk of off-target effects and bleeding complications commonly associated with conventional anticoagulants. Moreover, complementary ‘antidote’ nucleic acid sequences can be used to rapidly reverse aptamer activity (as for example NCT00715455). While the clinical efficacy of these aptamer/antidote systems in septic coagulopathy remains untested, they offer a promising strategy to further reduce side effects such as bleeding complications.

Beyond their therapeutic potential, aptamers show strong promise as diagnostic and prognostic tools through real-time monitoring of biomarkers such as procalcitonin, fibrinogen, neutrophil elastase, or protein C levels. Aptamer-based biosensors [129] in particular, could enable rapid and accurate detection of sepsis and its complications, provided that biomarker relevance in septic coagulopathy is further validated.

## Perspectives

Aptamers possess a distinctive set of theoretical and practical advantages, including high target specificity, negligible intrinsic toxicity, reversible activity through complementary antidote sequences, and tuneable pharmacodynamic properties. Despite these advantages, their application in the management of sepsis-induced coagulopathy remains at an early stage, and further comprehensive investigations are required before clinical implementation can be envisaged.

In particular, the establishment of standardized methodologies to assess their therapeutic efficacy will be essential for successful clinical translation. In addition to monitoring the evolution of DIC scores, the efficiency of aptamers cited in this review could be evaluated by measuring soluble markers responsible for endothelial activation, plasma clotting times, markers of fibrinolysis such as plasminogen, D-dimers and fibrin monomers, and plasma activated fragments of complement. Nevertheless, establishing the optimal window for evaluating aptamer efficacy in patients will be a critical step toward clinical validation.

Future research should also aim to confirm the specificity, safety and efficacy of aptamer-based interventions in relevant clinical settings and to evaluate their potential integration into precision-medicine strategies for sepsis-induced DIC. As mechanistic insights into thrombo-inflammatory pathways continue to advance, aptamers may emerge as valuable modulators within a more targeted and individualized therapeutic framework. Notably, their relatively short *in vivo* half-life, often regarded as a limitation, may confer an advantage in sepsis and septic shock, where rapid and reversible modulation of excessive coagulation may be desirable.

In conclusion, the versatility, high affinity, and chemical adaptability of aptamers make them emerging candidates for both therapeutic intervention and point-of-care diagnostics in critical care. Although they remain a comparatively underexplored class of innovative nucleic acid therapeutics [127], the breadth of potential targets described in this review, and the flexibility of aptamer design provide a promising foundation for innovation in the treatment of sepsis-related coagulopathies.

## CRediT authorship contribution statement

Conceptualization and structure, M.M., J.H. and L.C.; formal analysis, M.M.; original draft writing, M.M.; supervision and project administration: J.H. and L.C. All authors have contributed to writing and reviewing. All authors have read and agreed to the submitted version of the manuscript

All the authors have contributed to writing and have revised the manuscript.

## Consent for publication

NA.

## Ethics approval and consent to participate

NA.

## Funding

This research was supported by ADIRAL.

## Declaration of competing interest

JH has received honoraria for lectures from Pfizer PFE France, Sanofi Aventis France, Inotrem, MSD, Octapharma and Shionogi and is part of the steering committee from Bayer and AngioDynamics. The other authors have no conflict of interest to declare.
